# Supplementary material for: Valproic Acid Inhibits Progressive Hereditary Hearing Loss in a KCNQ4 Variant Model through HDAC1 Suppression
Source: Int J Mol Sci. 2023 Mar 16;24(6):5695. doi: 10.3390/ijms24065695 (PMC10058529; doi:10.3390/ijms24065695)
Supplement: Supplementary file 1 [file ijms-24-05695-s001.zip › SUPPLEMENT FIGURE LEGENDS v2.pdf]

SUPPLEMENT FIGURE LEGENDS

Supplementary Figure S1.

A. Representative image of 7 weeks KCNQ4 +/+ mice cochlea immunostaining. Whole-mount immunostaining of the cochlea with antibodies directed toward KCNQ4 (green) and Prestin (red) to show the KCNQ4 cochlea expression. Nuclei are stained with DAPI (blue). Scale bar: 25 μm

Supplementary Figure S2.

A. KCNQ4 KI/KI variant-induced hearing loss as shown by click sound auditory brainstem response (ABR). **P<0.01, ***p<0.001, Numerals in bar graphs are the numbers of samples. Error bars represent S.E.M.

B. The tone burst ABR results showed a significantly increased at 8, 16, 24, and 32 kHz in KCNQ4 KI/KI variant mice. **P<0.01, ***p<0.001, Numerals in bar graphs are the numbers of samples. Error bars represent S.E.M.

Supplementary Figure S3.

A. KCNQ4 KI/KI variant-induced hearing loss protective effect by valproic acid (VPA) delivered via osmotic pump. Hearing threshold checked by auditory brainstem response (ABR) using click sound. *P<0.05, **p<0.01. Error bars represent S.E.M (N=4).

B. The tone burst ABR results showed a significantly decreased when 5 and 6 weeks at 8 kHz in KCNQ4 KI/KI variant by implanted with an osmotic pump delivering VPA treated mice. *P<0.05. Error bars represent S.E.M. (N=4)

Supplementary Figure S4.

A. Endogenous KCNQ4 and HSP90β reduced protein expression by transient transfection of *pCs2+-3myc-HDAC1* however *pCs2+-3myc-HDAC2* did not influence protein expression in HEK293T cells.

B. Endogenous KCNQ4 and HSP90β did not protein expression change by transient transfection of *pcDNA3.1-HDAC3-Flag* and *pAP3neo-HDAC4-Flag* in HEK293T cells.
