# Supplementary figures and images for: Valproic Acid Inhibits Progressive Hereditary Hearing Loss in a KCNQ4 Variant Model through HDAC1 Suppression
Source: Int J Mol Sci. 2023 Mar 16;24(6):5695. doi: 10.3390/ijms24065695 (PMC10058529; doi:10.3390/ijms24065695)

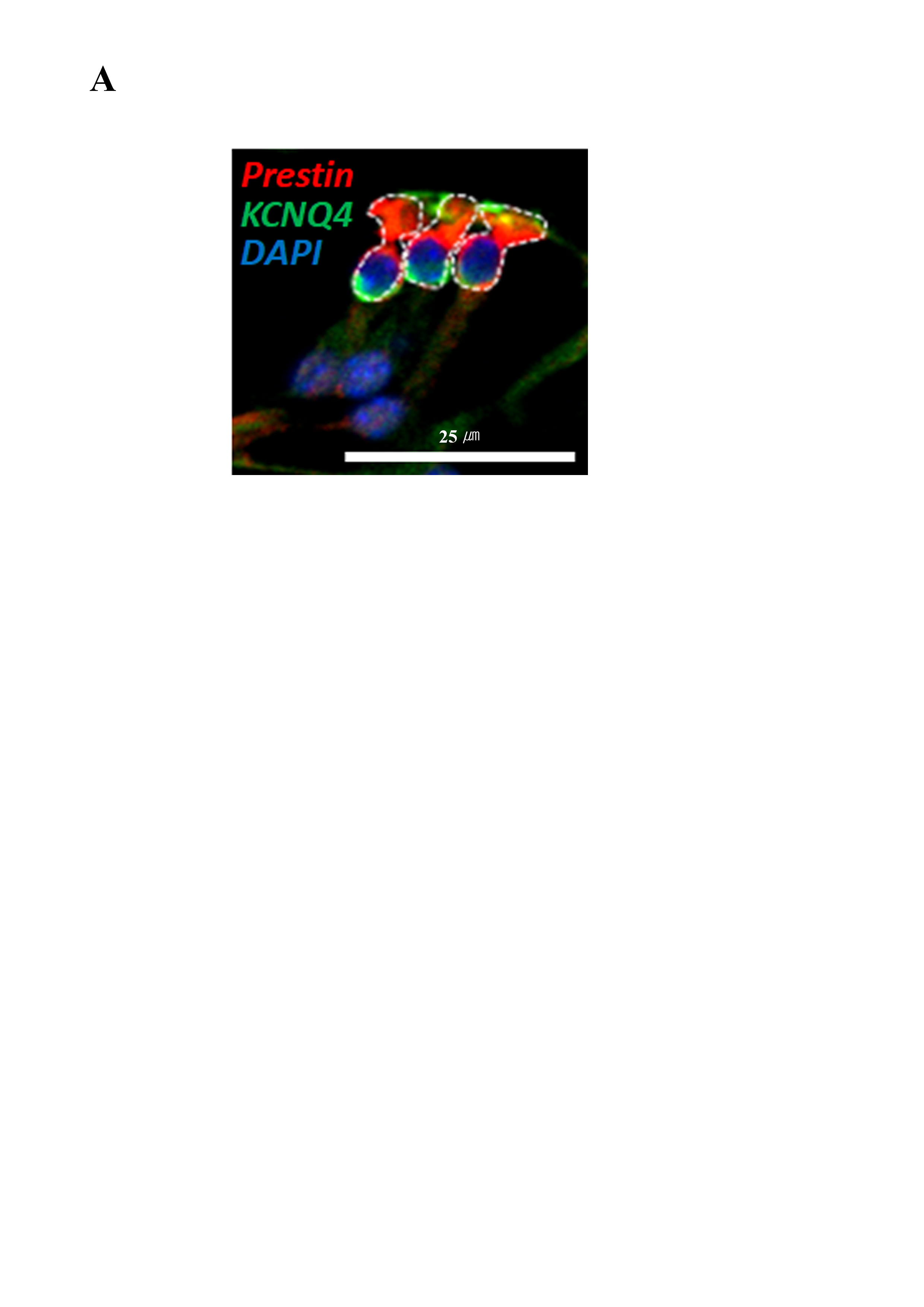

Supplement: Supplementary file 1 [file ijms-24-05695-s001.zip › Supplement Figure S1.jpg]

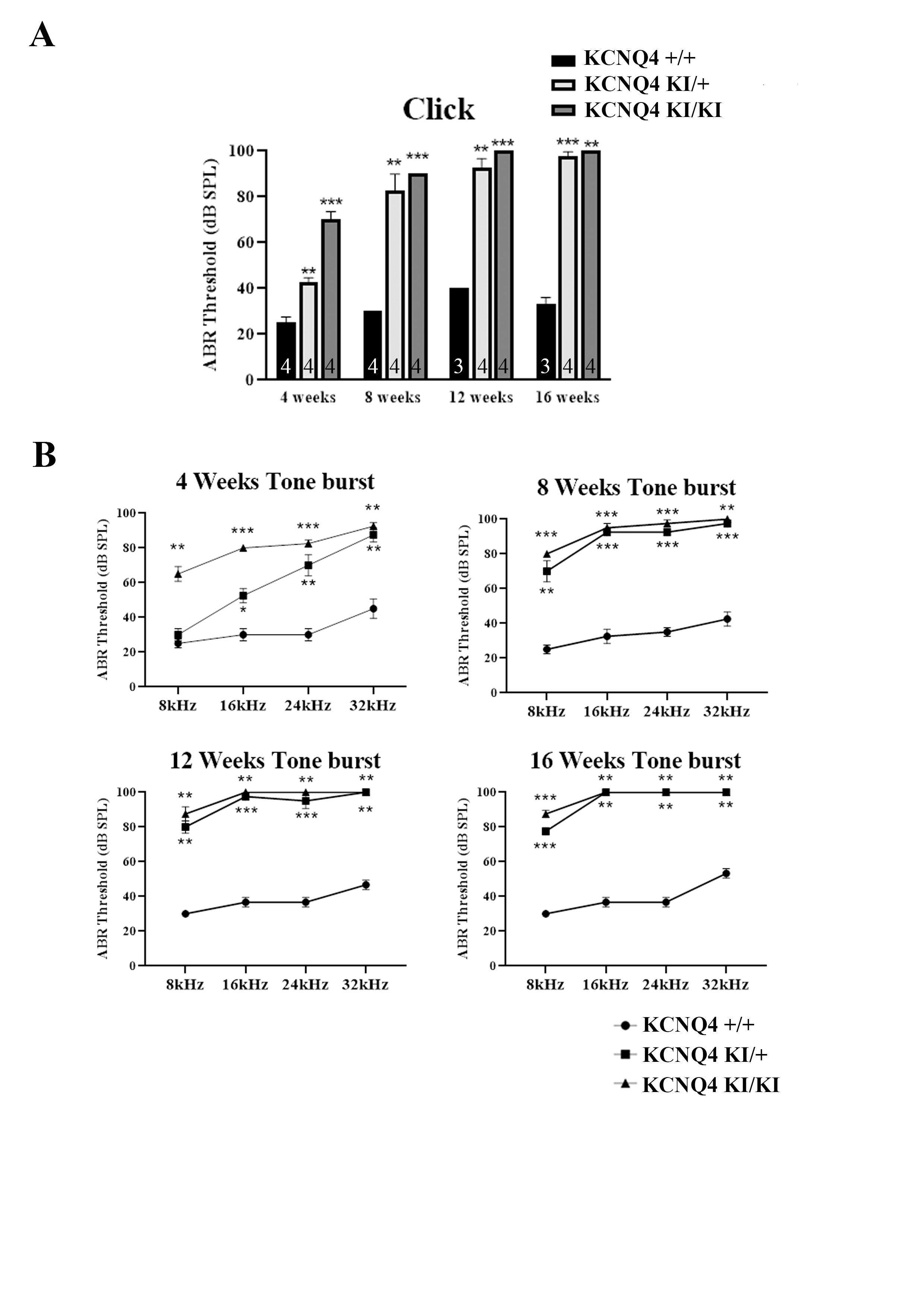

Supplement: Supplementary file 1 [file ijms-24-05695-s001.zip › Supplement Figure S2.jpg]

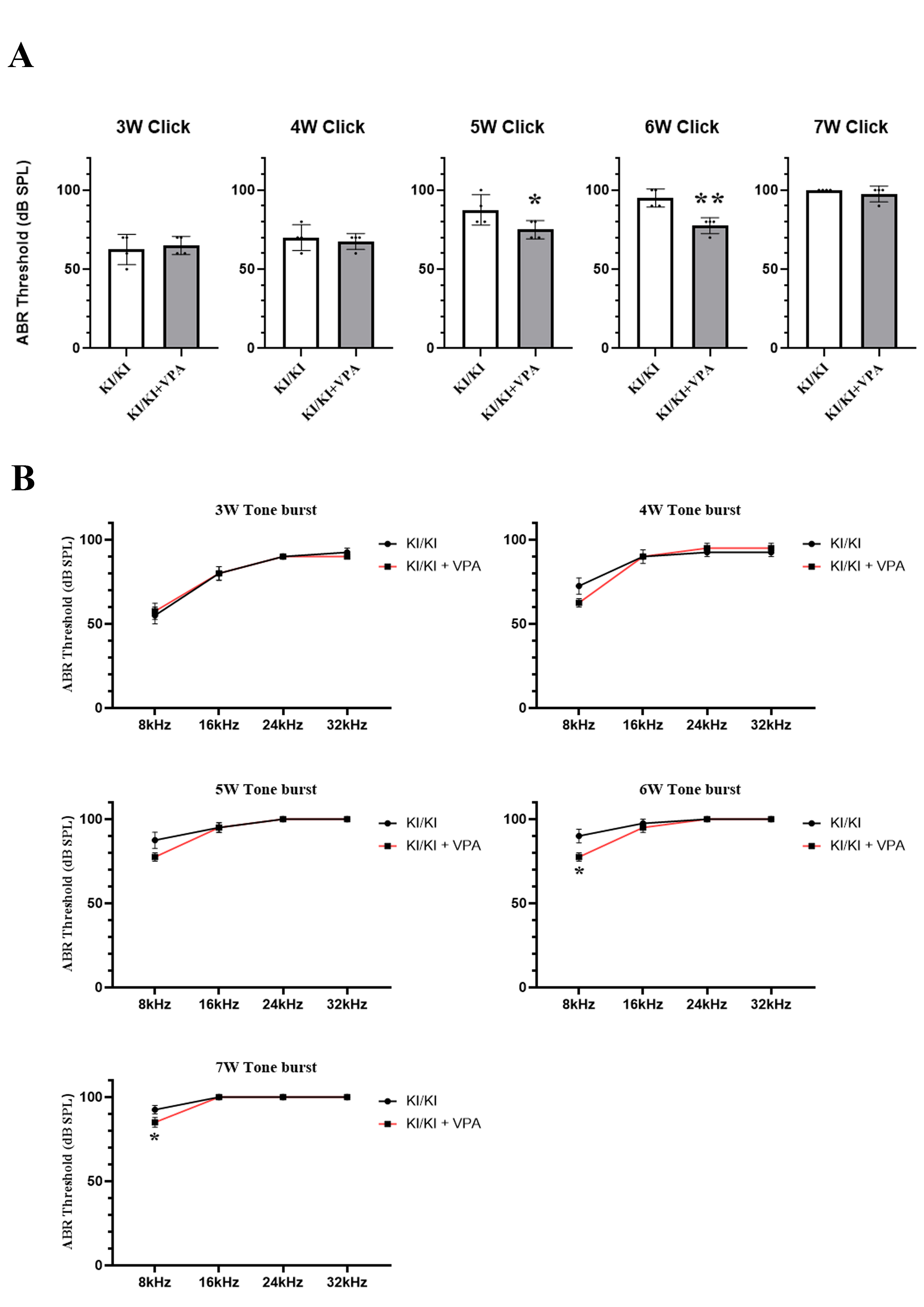

Supplement: Supplementary file 1 [file ijms-24-05695-s001.zip › Supplement Figure S3.jpg]

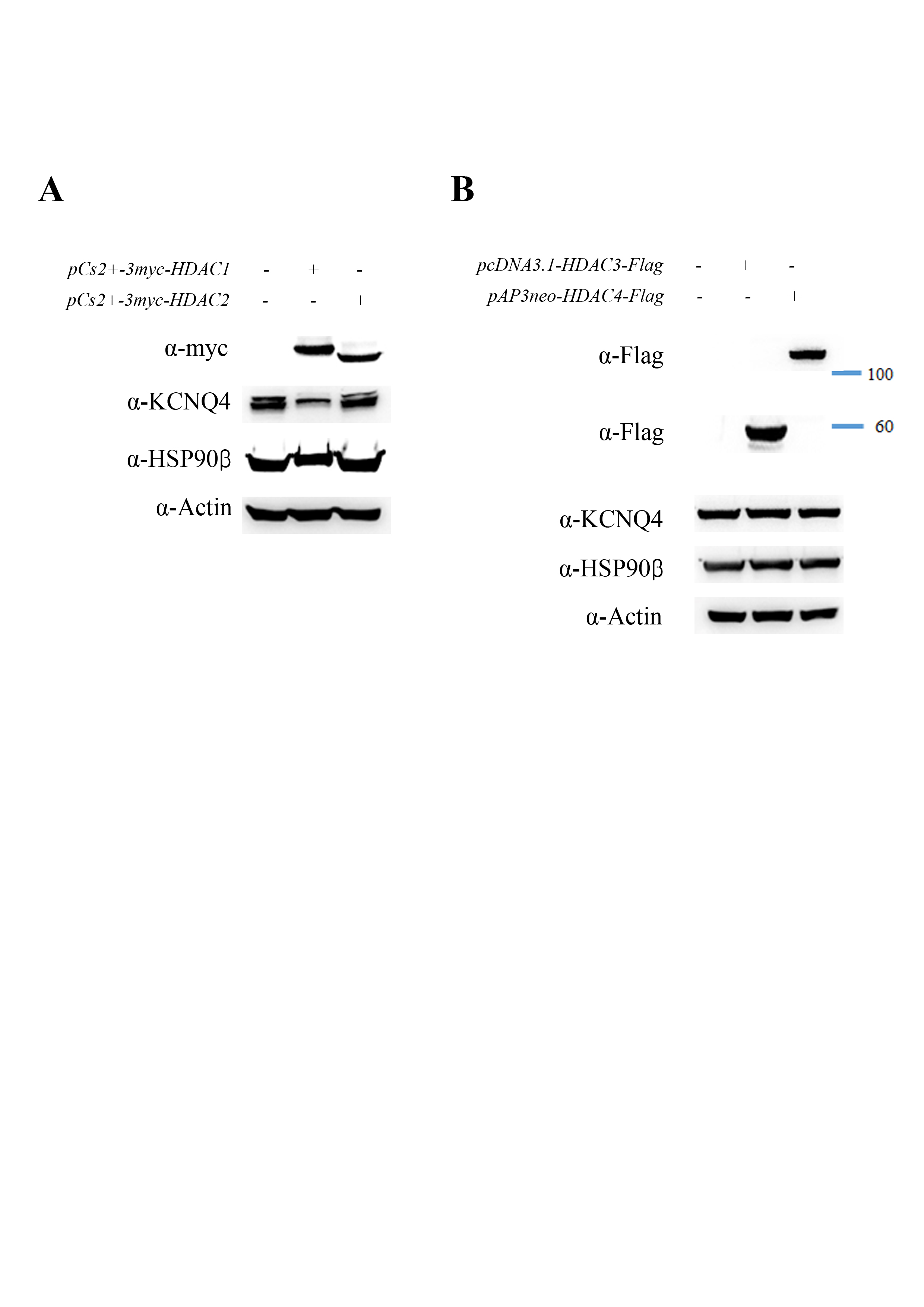

Supplement: Supplementary file 1 [file ijms-24-05695-s001.zip › Supplement Figure S4.jpg]
